# Positive and Negative Regulatory Mechanisms for Fine-Tuning Cellularity and Functions of Medullary Thymic Epithelial Cells

**DOI:** 10.3389/fimmu.2015.00461

**Published:** 2015-09-08

**Authors:** Taishin Akiyama, Ryosuke Tateishi, Nobuko Akiyama, Riko Yoshinaga, Tetsuya J. Kobayashi

**Affiliations:** ^1^Division of Cellular and Molecular Biology, Institute of Medical Science, University of Tokyo, Tokyo, Japan; ^2^Institute of Industrial Science, University of Tokyo, Tokyo, Japan

**Keywords:** medullary thymic epithelial cells, autoimmune disease, negative feedback, mathematical modeling, T cells, thymus

## Abstract

Self-tolerant T cells and regulatory T cells develop in the thymus. A wide variety of cell–cell interactions in the thymus is required for the differentiation, proliferation, and repertoire selection of T cells. Various secreted and cell surface molecules expressed in thymic epithelial cells (TECs) mediate these processes. Moreover, cytokines expressed by cells of hematopoietic origin regulate the cellularity of TECs. Tumor necrosis factor (TNF) family RANK ligand, lymphotoxin, and CD40 ligand, expressed in T cells and innate lymphoid cells (ILCs), promote the differentiation and proliferation of medullary TECs (mTECs) that play critical roles in the induction of immune tolerance. A recent study suggests that interleukin-22 (IL-22) produced by ILCs promotes regeneration of TECs after irradiation. Intriguingly, tumor growth factor-β and osteoprotegerin limit cellularity of mTECs, thereby attenuating regulatory T cell generation. We will review recent insights into the molecular basis for cell–cell interactions regulating differentiation and proliferation of mTECs and also discuss about a perspective on use of mathematical models for understanding this complicated system.

## Introduction

Thymic epithelial cells (TECs) are essential for T cell development and self-tolerance induction in the thymus (1). TECs are classified into medullary TECs (mTECs) and cortical TECs (cTECs) according to their localizations in the thymus. While cTECs mainly support the early differentiation and proliferation of T cells and positive selection of self-MHC-restricted T cells, several lines of evidence indicate critical roles of mTECs in preventing the onset of autoimmune diseases in human and mice (2). mTECs uniquely express many kinds of tissue-specific self-antigens (TSAs) (2–4). mTECs expressing high levels of MHC class II and co-stimulatory molecules, such as CD80, would be capable of directly presenting these TSAs to medullary T cells (5). Alternatively, TSAs in mTECs are transferred to thymic dendritic cells (DCs) and indirectly presented to T cells (6, 7). When T cells recognize these presented TSAs through high avidity interactions, they undergo apoptosis or are converted into regulatory T cells (Tregs) (2). Expression of TSAs is, in part, regulated by nuclear protein autoimmune regulator (AIRE), and dysfunctional mutations in AIRE provoke autoimmune diseases in humans (3, 8, 9). In addition to such roles in preventing autoimmunity, recent studies suggest that immune tolerance to some tumors might be under the control of mTECs (10–12). Therefore, understanding cellular and molecular mechanisms to regulate mTEC differentiation, proliferation, and apoptosis is an important issue.

Recent studies have revealed new aspects of mTEC differentiation and proliferation (13–15). One recent study indicated a new mechanism that promotes recovery of TECs following thymic injury induced by γ-irradiation (13). In addition, molecular mechanisms of negative regulation in mTEC differentiation have been reported (14, 15). In this review, we will summarize these new findings. Moreover, these new findings together with previous studies imply the existence of considerably more complicated cellular and molecular mechanisms regulating mTEC cellularity than was previously recognized. Such a sophisticated system can ensure precise regulation of TEC functions in T cell differentiation, selection, and tolerance induction. Therefore, we also present our perspectives on how mathematical modeling might contribute to understanding regulation of TEC cellularity and functions.

## Positive Regulatory Mechanisms for mTEC Differentiation and Regeneration

### Tumor necrosis factor receptor family signaling and NF-κB pathways in mTEC differentiation

Medullary TECs constitute a heterogeneous cell population under constant differentiation (1). The roles of tumor necrosis factor (TNF) receptor family signaling on mTEC differentiation were previously summarized (16). Therefore, we briefly mention this topic. Receptor activator of NF-κB (RANK), CD40, and lymphotoxin β-receptor (LtβR), and all members of the TNF receptor family, have been reported to promote mTEC differentiation. RANK signaling appears to play a dominant role in the differentiation of mTECs expressing Aire (16–19). The role of CD40 may be similar to that of RANK in the postnatal period because deletion of both RANK and CD40 signaling resulted in almost complete loss of Aire-expressing mTECs, as compared to a partial reduction of mature mTECs by the absence of either RANK or CD40 signaling (16). In the embryonic thymus, only RANK signaling is active (18) because thymic expression of CD40 ligand starts in the perinatal period (20). LtβR might control several distinct steps in mTEC differentiation (16, 21–23). An early study suggests that LtβR signaling induces Aire expression (24). However, later studies did not support the direct connection between LtβR signaling and Aire expression (25, 26). This apparent discrepancy remains to be solved. Moreover, some studies revealed that the absence of LtβR signaling causes a disturbance in three-dimensional organization of mTECs (27, 28), suggesting a distinct role of LtβR signaling from other TNF receptor signaling.

Ligands of these TNF receptor family members are expressed mainly in cells of hematopoietic origin. Previous studies revealed that RANK ligand (RANKL) is expressed in innate lymphoid cells (ILCs) and positively selected CD4^+^CD8^−^ T cells (CD4SP) (16, 19). Moreover, RANKL expression was detected in fractions of CD4^−^CD8^−^ thymocytes (double negative, DN) and γδT cells (29, 30). Conditional deletion of RANKL from each cell type would be needed to identify what types of cells are major sources of the RANKL for mTEC differentiation unambiguously. CD40 ligand is most highly expressed in CD4SP (19, 31). The ligand of LtβR is a heterotrimer consisting of secreted lymphotoxin α and membrane bound lymphotoxin β. Expression of lymphotoxin β appears to be high in CD4SP, CD8SP, and ILCs (19, 25, 32). Consequently, interactions with these cells might be required for differentiation of mTECs.

Signaling by RANK, CD40, and LtβR activates the transcription factor NF-κB. The NF-κB family consisting of five members (i.e., RelA, RelB, c-Rel, p105/p50, and p100/p52) that form hetero- and homodimers and are sequestered in the cytosol typically by binding to their inhibitor protein IκBs in unstimulated cells. Signal-dependent degradation of IκBs by the ubiquitin–proteasome pathway results in the translocation of NF-κB into the nucleus, which in turn promotes the expression of genes controlling various cellular responses (33). RANK, CD40, and LtβR signaling are capable of activating two distinct NF-κB pathways: the canonical NF-κB pathway and the non-canonical NF-κB pathway (16, 33). Briefly, activation of the canonical NF-κB pathway leads to nuclear translocation of RelA or c-Rel bound to p50. On the other hand, the non-canonical NF-κB pathway results in nuclear translocation of RelB bound to p52.

RelB deficiency and a dysfunctional mutation of NF-κB inducing kinase (NIK), a signal transducing molecule of the non-canonical NF-κB pathway, cause a severe reduction in the number of mature mTECs (34–37), suggesting an essential role for the non-canonical NF-κB pathway in mTEC differentiation. TNF receptor-associated factor 6 is a signal transducer of the canonical NF-κB pathway and is also essential for the mTEC differentiation (38). These data imply that roles of the canonical and non-canonical NF-κB pathways are not redundant, but that both are essential for mTEC differentiation. Elucidation of the functional differences between these two NF-κB pathways in mTEC differentiation remains to be determined.

### Role of IL-22 signaling in the regeneration of TECs

Many stressors, such as psychological stress, virus infection, chemotherapy, and irradiation in bone marrow transplantation therapy, provoke acute thymic involution in which TECs and thymocytes rapidly decrease (39, 40). Although recovery from these acute thymic injuries usually occurs, incomplete recovery of thymic cells can increase the risk of immunodeficiency and autoimmunity (39, 40). Therefore, understanding the molecular and cellular mechanisms of acute thymic involution and its recovery is necessary. A recent study revealed a critical role for IL-22 in the repair of TECs after thymic involution induced by radiation (13).

IL-22 is reportedly produced by T helper 1 (Th1) cells, Th17 cells, and Th22 cells (41, 42). In addition, group 3 ILCs secret high amounts of IL-22 (41, 42). The IL-22 signal is transmitted through the IL-22 receptor (IL-22R), consisting of IL-22R1 and IL-10R2 subunits, and activates various downstream signaling (41). The IL-22 signal promotes the regeneration of epithelial cells in the liver, airway, and intestine after injury (42).

Dudakov et al. (13) reported that expression of IL-22 is upregulated in the thymus after total body irradiation in mice, which may mimic thymic injury by radiation therapy for malignant leukemia. IL-22 expression showed an inverse correlation with changes in thymic cell numbers after the irradiation. They further demonstrated a delay of TEC recovery in IL-22-deficient mice (*Il22*^−/−^) after the irradiation. Because thymic cell numbers were not reduced in the thymus of untreated *Il22*^−/−^ mice, IL-22 appears to function in the regeneration of TECs specifically. They also determined that lymphoid tissue inducer (Lti), which belongs to group 3 ILCs, was the producer of IL-22 in this context. IL-22 expression in the Lti was induced by signaling of IL-23 secreted from thymic DCs. The reduction of CD4^+^CD8^+^ thymocytes (double positive, DP) by irradiation triggered the secretion of IL-23 from DCs, although the molecular mechanism remains unclear. IL-22 appears to enhance proliferation of mTECs as well as cTECs. Because Aire-positive mTECs are reportedly post-mitotic (43), it should be determined in the future whether IL-22 signaling alone is capable of inducing the proliferation of Aire-positive mTECs in this situation or whether other signals are necessary for the recovery of Aire-positive mTECs.

## Negative Regulatory Mechanisms for Fine-Tuning the Cellularity of mTECs

### Negative regulation of mTEC cellularity by TGF-β signaling

Tumor growth factor (TGF)-β has diverse functions during the development and homeostasis of various tissues (44). Binding of TGF-β to its cell surface receptor complex, consisting of type II receptors (TGF-β RII) and the type I receptors (TGF-β RI), induces activity in its cytoplasmic serine/threonine kinases, thereby activating Smad protein complex. The activated Smad complex is subsequently translocated into the nucleus and promotes gene expression. As a result, TGF-β signaling induces anti-proliferative and pro-apoptotic effects in many types of cells.

In the thymus, TGF-β is expressed by cTECs and immature thymocytes (45, 46). On the other hand, the TGF-β receptor complex is expressed in both cTECs and mTECs (47). Hauri-Hohl et al. have recently reported a role for TGF-β signaling in regulation of mTEC number (14). They prepared mice lacking TGF-β RII expression specifically in TECs. Interestingly, the cellularity of only mTECs was increased by the lack of TGF-β signaling in TECs. Consistently, administration of a TGF-β RI inhibitor also increased the mTEC number. Thus, TGF-β signaling limits mTEC cellularity selectively, although both mTEC and cTECs should receive the signals.

The limitation of mTEC number by TGF-β signaling is less likely due to its anti-proliferative and pro-apoptotic effects. Instead, *in vitro* data have suggested that TGF-β signaling interferes with the activation of non-canonical NF-κB signaling; the mechanism, however, remains unclear. Given that RANK, CD40, and LtβR signaling all activate non-canonical NF-κB signaling, it is possible that TGF-β inhibits non-canonical NF-κB signaling triggered by these receptors, thereby limiting the mTEC cellularity. This idea might explain the mTEC-selective inhibition by TGF-β.

### Regulation of gene expression and differentiation of mTECs by the ETS family member Spi-B

**Figure 1:**
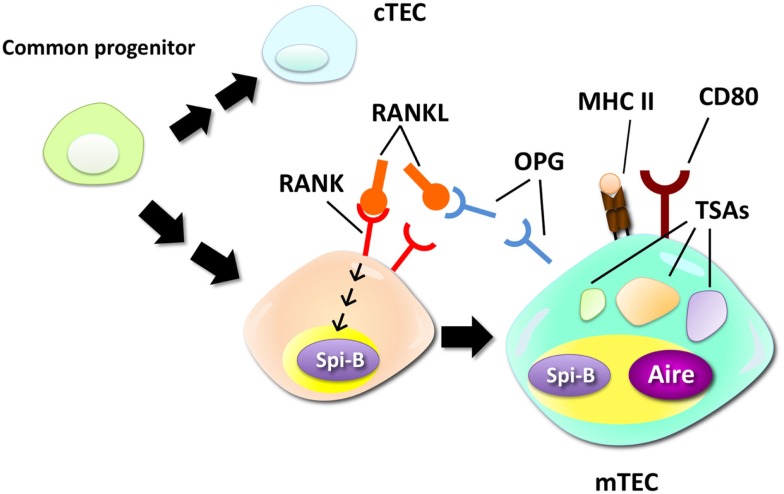
**Negative regulation of mTEC differentiation by the RANK–Spi-B–OPG–RANKL feedback loop**. mTECs are derived from a common progenitor that can give rise to both mTECs and cTECs. RANK signaling promotes differentiation of relatively immature mTECs into mTECs expressing high levels of MHC class II (MHC II), CD80, and Aire. A recent study suggested that RANK signaling upregulates expression of Spi-B. Spi-B promotes expression of some TSAs, CD80, and osteoprotegerin (OPG), a secreted decoy receptor for RANK, in mTECs. OPG, in turn, competitively inhibits RANKL–RANK interactions, thereby inhibiting the RANKL-dependent process of mTEC differentiation.

The Ets family transcription factor Spi-B has been recently identified as a regulator of mTEC differentiation (15). RANKL signaling rapidly upregulates Spi-B expression in *in vitro* thymic stromal culture via the NIK-dependent NF-κB pathway. Lack of Spi-B caused an increase in the number of mTECs expressing high levels of MHC II. On the other hand, expression of co-stimulatory molecule CD80, CD86, and some of TSAs in mTECs were strikingly reduced in Spi-B-deficient (*Spib*^−^*^/^*^−^) mTECs. Thus, Spi-B apparently has dual functions in mTEC differentiation: Spi-B limits the number of mature mTECs and promotes some mTEC-functional genes. In addition, expression of osteoprotegerin (OPG), a decoy receptor of RANKL (48), was significantly reduced in *Spib*^−^*^/^*^−^ mTECs. OPG was previously reported to be a negative regulator of mTEC differentiation by inhibiting RANKL signaling (19). Moreover, the Spi-B-mediated limitation of mTEC cellularity was not detected in the absence of OPG. These facts suggest that Spi-B induced by RANK signaling upregulates OPG expression in mTECs, thereby competitively inhibiting the RANKL signal-inducing mTEC differentiation (Figure 1). Thus, this negative feedback regulation finely tunes the cellularity of mTECs. Noticeably, negative regulation of mTEC differentiation by the Spi-B–OPG axis starts in the peri- to neonatal period, during which Aire mediates long-lived tolerance (49, 50).

### Biological and physiological significance of negative regulation of mTEC cellularity

The effect of TGF-β signaling in mTECs on thymic T cell differentiation was investigated (14). The number of SP thymocytes and the frequency of CD4SP were mildly increased by the absence of TGF-β signaling. Moreover, export of thymic T cells to the periphery was delayed in the postnatal period of these mice. These data suggest that the increase in mTEC number prolongs the dwelling time of mature T cells in the thymic medulla. The absence of TGF-β signaling in TECs resulted in an increase in thymic Tregs and their precursors and a reduction in the frequency of thymic and peripheral Th17 cells.

Osteoprotegerin-deficient (*Opg*^−/−^) mice were used to investigate the role of the negative feedback circuit consisting of RANKL–Spi-B–OPG in thymic T cell selection (15). The OPG deletion in the thymic stroma led to an increase in the number and frequency of Tregs and Treg precursors in the thymus. Together with the findings on TGF-β-mediated TEC regulation, this suggested that negative regulation of mTECs attenuates the generation of thymic Tregs. Importantly, the increase in Treg generation by the deletion of OPG initiates in the perinatal period. A recent study revealed that Tregs generated during this period are functionally distinct from those produced in the adult thymus and that these Tregs play a critical role in long-lived tolerance induction (49, 50). Therefore, fine-tuning in the generation of Tregs during this period by this negative feedback loop could have an impact on T cell tolerance in adults.

What is the physiological impact of these negative regulations? Suppose that mTECs simply played a role in preventing the onset of autoimmune disease by negative selection and conversion of Tregs. In this case, the inhibitory regulations of mTEC differentiation might be harmful to the body. Indeed, the absence of TGF-β signaling in TECs attenuates autoimmunity caused by a chronic ablation of Tregs (14), suggesting a reduction of self-tissue reactive T cells by abolishing TGF-β-mediated negative regulation. Thus, this finding supports the idea that negative regulation of mTECs would increase the risk of autoimmunity. Besides the critical role of mTECs in inducing tolerance toward various self-tissues, recent studies have shown that mTECs promote T cell tolerance to tumors (10–12). The roles of RANKL–Spi-B–OPG negative feedback regulation in tumor immunity were tested. When this negative feedback loop was abolished by OPG depletion in the thymic stroma, tumor growth, and incidence of carcinogenesis were increased (14). These findings suggest that this negative feedback regulation might promote tumor immunity and optimize the trade-off between prevention of autoimmunity and induction of tumor immunity. Thus, negative regulation of mTEC number may contribute to immune responses toward self-antigens in tumors that are originally derived from self-tissues.

## Perspective on Mathematical Modeling for TEC Cellularity

As described above, many cell types and molecules are involved in the regulation of mTEC cellularity. Consequently, T cell selection and tolerance induction supported by mTECs could be finely tuned by a combination of various mechanisms under steady state and pathological condition. Mathematical modeling would help us to understand this complicated situation. However, mathematical modeling on dynamics of TECs, including mTECs, has not been reported yet. On the other hand, there are several studies on mathematical modeling of thymocyte development. The similar mathematical approach as that used for investigations into thymocyte development can be employed for TEC development. Moreover, because interactions with thymocytes are critical for differentiation, proliferation, and survival of TECs, dynamics of thymocytes should be included in the mathematical modeling of TECs. In this section, we briefly discuss about a perspective on use of mathematical models to understand dynamics of TEC population by referring to previous mathematical modeling studies on thymocytes.

Generally, tracking cell fates over time at the single-cell level is experimentally demanding and almost impossible *in vivo*. Therefore, mathematical models are indispensable to extract biologically relevant information on cellular dynamics and differentiation from population-level measurements. As more detailed information is obtained by new experimental methods, the mathematical models have also evolved from simple ordinary differential equations (ODEs) to cellular automata, compartment models, and stochastic models in order to account for different subtypes of lymphocytes, their cellular heterogeneity, and spatial niches that they reside.

Although dynamics of thymocyte populations were modeled by their types, i.e., DN, DP, and SPs, the interactions of thymocytes with TECs were not explicitly incorporated in many models (51–55). In order to investigate the contributions of cortical and medullary selection, the influence of the TECs and the thymic environment were incorporated more explicitly into models in other studies. Fano et al. modeled the interaction of the thymocytes with the cortical and medullary APCs to estimate the fractions of the positively and negatively selected thymocytes in the cortex and the medulla in relation to the diversity of presenting ligands (56). In other studies (57, 58), the anatomical structure of the thymus, together with cell types, was incorporated explicitly into an investigation of the interrelation between thymocyte migration and selection. In these studies, however, the TECs were considered to be in a static thymic environment. In reality, the TECs also differentiate and proliferate homeostatically in the thymus.

Influence of the thymocyte dynamics should be incorporated into the mathematical modeling of TEC development because not only intra- but also inter-regulation of thymocytes and TECs is quite important when we consider the differentiation process of TECs and recovery of TECs from damage and its involution by aging, in which both thymocytes and TECs change their population dynamically (59, 60). Thus, we think that mathematical models will be crucial for understanding the joint dynamics of thymocytes and TECs by disentangling their complicated cell–cell interactions.

## Concluding Remarks

Several types of cells and various positive and negative signaling pathways appear to control the cellularity of mTECs under physiological and pathological condition. Because cellular development and recovery from injuries are time-dependent processes, these mechanisms should be regulated in a precise and timely manner. Employment of mathematical modeling is a promising approach to understand these temporally regulated processes in the future.

## Conflict of Interest Statement

The authors declare that the research was conducted in the absence of any commercial or financial relationships that could be construed as a potential conflict of interest.
